# The NMDAR modulator NYX-2925 alleviates neuropathic pain via a Src-dependent mechanism in the mPFC

**DOI:** 10.1016/j.ynpai.2019.100039

**Published:** 2019-12-04

**Authors:** Gladys Morrison, Marina N. Asiedu, Jessica M. Priebe, Jacqueline Dunning, Nayereh Ghoreishi-Haack, Roger A. Kroes, M. Scott Bowers, Amanda L. Barth, Cassia N. Cearley, Joseph R. Moskal

**Affiliations:** a Aptinyx Inc., Evanston, IL, United States; b Falk Ctr. for Mol. Therapeutics, McCormick School of Engineering, Northwestern University, Evanston, IL, United States

**Keywords:** Neuropathic pain, Medial prefrontal cortex, NMDAR's, Src kinase, Chronic Constriction injury (CCI)

## Abstract

Previous studies have shown that oral administration of the NMDAR modulator NYX-2925 alleviates pain in several animal models of neuropathic pain and this appears to be through mPFC, but not spinal, mediated mechanisms. While much is known about the impact of neuropathic pain on NMDAR-mediated signaling in the spinal cord, limited studies have focused on the brain. In the current study, we assess signaling changes associated with NMDAR-mediated plasticity in the mPFC and the impact of NYX-2925 administration on the normalization of these signaling changes. We found a decrease in activated Src levels in the mPFC of animals with chronic constriction injury (CCI) of the sciatic nerve. While Src mediated activation of NMDARs was also decreased in CCI animals, the main NMDAR phosphorylation site of CAMKII was not affected. This is in opposition to what has been found in the spinal cord, where both Src and CAMKII activation are increased. Oral administration of NYX-2925 restored levels of activated Src and Src phosphorylation sites on GluN2A and GluN2B in the mPFC, with no effect on activated CAMKII levels. The analgesic effect of NYX-2925 appears dependent on this restoration of Src activation in the mPFC, as co-administering Src activation inhibitors prevented the NYX-2925 analgesic effect. Overall, these data suggest that NMDAR-mediated signaling plays a key role in neuropathic pain, albeit in different directions in the spinal cord vs. the mPFC. Furthermore, the analgesic effect of NYX-2925 appears to involve a restoration of NMDAR-mediated signaling in the mPFC.

## Highlights

• Direct injection of the NMDAR modulator NYX-2925 in the mPFC, reverses allodynia.
• Src activity is downregulated in the mPFC of a CCI neuropathic pain model.
• NYX-2925 restores pain induced decrease of phosphorylated NMDAR’s.
• NYX-2925 restores pain induced decrease of Src-mediated intracellular signaling.

## 1 Introduction

Chronic neuropathic pain is a disease resulting from long-term changes to synaptic plasticity mechanisms in the spinal cord (Costigan et al., 2009, Woolf and Salter, 2000), as well as in subcortical and cortical areas (Zhuo, 2008, Zhuo, 2018). Of the cortical regions affected, the mPFC is a critical region known to regulate chronic pain with several studies showing that it undergoes structural, morphological, and functional reorganization in neuropathic pain (Alvarado et al., 2013, Bushnell et al., 2013, Kelly et al., 2016, Lee et al., 2015, Metz et al., 2009). Preclinical studies have shown overall reduced activity of the prelimbic mPFC in neuropathic pain that appears to result from impaired glutamatergic transmission (Kelly et al., 2016). This reduction in mPFC activity is also implicated in affective and cognitive changes associated with chronic pain (Millecamps et al., 2007, Wang et al., 2015). The discovery of novel compounds that can regulate mPFC activity after pain has become centralized may be important in the treatment of neuropathic pain.

Recent work has shown that NYX-2925, an N-methyl-D-aspartate receptor (NMDAR) modulator acts as a glutamate co-ligand to facilitate synaptic plasticity. Furthermore, it has also been shown to regulate levels of GluN2A and 2B at the synapse (Bowers et al., 2019, Khan et al., 2018). Additionally, it enhances learning and memory, and has therapeutic potential for the treatment of neuropathic pain (Ghoreishi-Haack et al., 2018, Khan et al., 2018). Interestingly, in the rat chronic constrictive injury (CCI) model of neuropathic pain, intrathecal injection of NYX-2925 had no therapeutic effect, while systemic administration or direct infusion of NYX-2925 into the mPFC alleviated neuropathic pain (Ghoreishi-Haack et al., 2018). These data suggested that NYX-2925 may be acting in the brain, with direct activity in the mPFC, to alleviate neuropathic pain.

A crucial signaling event in pain induced NMDAR-dependent enhancement of synaptic plasticity is the trafficking and stabilization of GluN2A and GluN2B to the cell surface, which is dependent on phosphorylation of NMDARs by intracellular kinases (Salter et al., 2009, Woolf and Salter, 2000). In neuropathic pain, the kinases Src and CAMKII are particularly important in driving (Chen and Roche, 2007, Liu et al., 2008, Qian et al., 2018, Salter et al., 2009) intracellular signaling cascades that converge to increase NMDAR plasticity via tyrosine phosphorylation of NMDAR subunits (Salter and Kalia, 2004, Yu et al., 1997). Disruption of these signaling cascades has been specifically implicated in the development and maintenance of neuropathic pain (Hildebrand et al., 2016, Nakazawa et al., 2001, Salter and Kalia, 2004).

In the present study we show that Src Family Kinase (SFK) -mediated signaling is disrupted in neuropathic pain-induced hypoactivity in the mPFC and NYX-2925 specifically normalizes the SFK-mediated proteins under conditions where it alleviates centralized neuropathic pain.

## 2 Methods

### 2.1 Animals

Adult (2–3 months old), male Sprague Dawley rats from Charles River Laboratory were used. Rats were housed (3 per cage) in Allentown IVC systems with Envigo Tek-Fresh bedding, supplemented with Enviro-Dri bedding (Shepherd Specialty Papers). All behavioral experiments were conducted within the light cycle (8 a.m. – 5p.m.). All studies were approved by the Northwestern University IACUC and in accordance with AALAC.

### 2.2 Chronic constriction injury (CCI) surgery

Rats were anesthetized using inhaled isoflurane (3% in O_2_, Novaplus, Irving, TX). CCI surgery was performed as previously described (Bennett and Xie, 1988, Ghoreishi-Haack et al., 2018). For the SHAM surgery, the sciatic nerve was exposed and left intact. Two- or 3-weeks post-surgery, baseline mechanical hypersensitivity of the ipsilateral hind paw was measured followed by oral dosing and testing 1 hr post oral dosing. For oral dosing, a stainless-steel feeding needle (Cadence Science) attached to 1 ml disposable syringe (Becton Dickinson) was introduced via the oral cavity into the lower oesophagus/stomach. The compound to be dosed was slowly expelled and the needle was slowly withdrawn. The compound was given in a volume calculated according to the individual rat body weight (2 ml/kg)

### 2.3 Intracranial cannula (ICC) surgery

Bilateral medial prefrontal cortex (mPFC) cannulation occurred immediately after CCI surgery. Using a Kopf stereotaxic device (California, USA), the rat’s skull was secured parallel to the frame under isoflurane anesthesia (3%, to effect). An incision was made through the skin of the skull between the eyes to the back of the ear and bregma was located. Stainless steel cannulae (14 mm, 26-gauge, Component Supply Company) were bilaterally implanted above the mPFC using the following coordinates from bregma (2.7 mm AP, ±1.03 mm ML, −3.5 mm DV from the skull surface, Paxinos & Watson). Cannulae were secured in a dental acrylic head cap via small screws. After surgery, obturators (14 mm, 33 gauge) were inserted into the cannulas and 3% H_2_O_2_ was locally applied around the head cap. Animals were housed one per cage immediately following surgery and thereafter for 14–20 days before behavioral testing.

To verify cannula placement after the completion of behavioral testing, rats were perfused, and the brains were further processed for histologic analysis. Brains were coronally sectioned at 100 µm with a cryostat (Leica, Buffalo Grove, IL) and Nissl-stained.

### 2.4 Medial prefrontal cortex (mPFC) microinjection

Twenty minutes prior to oral dosing of NYX-2925 (Sai Life Sciences, Maharashtra, India), as previously described (Barry and McGinty, 2017), 0.5ul of 10 µM 4-amino-5-(chlorophenyl)-7-(t-butyl)pyrazolo[3,4-D]pyrimidine (PP2 (a widely used, but non-selective Src family kinase (SFK) activation inhibitor); Selleck Chemicals, Houston, TX), 10 µM Compound 4 (KB SRC 4 (a specific Src activation inhibitor); Tocris, Minneapolis, MN) or 0.1% DMSO in double filtered PBS was bilaterally infused into the mPFC via an infusion pump and 1 μL Hamilton (Reno, NV) syringe connected to polyethylene tubing (PE20, Becton Dickinson). The attached micro injector was shaped to fit into the cannula and inject 1 mm below cannula. Infusions (0.25 μL/min) were administered over 2 min, and the micro injectors (33G) were left in the cannulae for an additional 2 min to allow for diffusion of the drug from the tip of the micro injector.

### 2.5 Mechanical hypersensitivity (Von Frey) testing

Rats were acclimated to the experimental room for 1hr and then habituated for an additional 10 mins to the testing chambers prior to beginning each testing session. As previously described (Chaplan et al., 1994, Chaplan et al., 1997), paw mechanical thresholds were determined by using the Dixon up-down method using von Frey filaments (North Coast Medical, Morgan Hill, CA). More details on von Frey was previously described (Ghoreishi-Haack et al., 2018). Any rat with paw withdrawal threshold (PWT) above 5 g at the baseline evaluation post CCI was excluded from the study (approximately 10%-15%).

### 2.6 Whole cell lysate and Immunoblotting assay

The mPFC from several rats in multiple groups (SHAM, CCI and CCI + 2925; *n*,9–12) were dissected and snap-frozen in liquid nitrogen. Immunoblotting was performed using the mPFC region contralateral to the CCI injury. Tissue samples were homogenized on ice in RIPA buffer mixed with Halt™ combined protease and phosphatase inhibitor cocktail (Thermo Scientific, Waltham, MA, USA). Protein lysates were collected and microcentrifuged at 12,000 rpm for 10 min at 4 °C. After centrifuging, supernatants were collected and protein concentration was assessed using Pierce BCA protein assay (Thermo Fisher, Waltham, MA). Samples were denatured with 4X laemmli buffer and 20% β-mercaptoethanol for 5 min at 75 °C. Equal concentrations of lysates (15μg) were separated by SDS-polyacrylamide gel electrophoresis and transferred to nitrocellulose membrane (Invitrogen, Waltham, MA). The blots were first stained with PonceauS to confirm uniform loading and transfer, followed by immunoblotting with the specific primary antibodies according to the manufacturer's instructions. Briefly, blots were blocked with 5% BSA in TBST for 1 hr at room temperature and then membranes were incubated with primary antibodies at dilutions as per the manufacturer's directions in 5% BSA overnight at 4 °C. Blots were then incubated with a horseradish peroxidase-linked secondary antibody for 1 hr, after which the labeled proteins were visualized by chemiluminescence (Amersham ECL Reagent) using a Bio-Rad ChemiDoc™ MP imaging System (Bio-Rad, Hercules, CA, USA). Gels were produced at least 2 independent times. All protein levels were quantified using Image-J Software (NIH) and normalized to GAPDH (protein levels/GAPDH levels).

### 2.7 Enriched synaptosome fraction

The prelimbic PFC from SHAM, CCI, and CCI rats dosed with (oral NYX-2925, 10 mg/kg, alone, or in combination with PP2 or Compound 4) were harvested and snap-frozen in liquid nitrogen. Tissue contralateral to the site of CCI injury and around the cannula tract were collected. Tissue samples were homogenized using a dounce homogenizer on ice in synaptic protein extraction reagent (Syn-PER^TM^ mixed with Halt™ combined protease and phosphatase inhibitor cocktail (Thermo Scientific, Waltham, MA, USA)). Protein lysates were collected, and micro centrifuged at 1200g for 10 min at 4 °C. After centrifuging, supernatants were collected recentrifuged again at 15,000g for 25 min. The supernatant, which makes up the cytosolic fraction was saved and the pellet, which makes up the enriched synaptosomes fraction was resuspended in SynPER reagent. Protein concentration for the enriched synaptosomes fraction was assessed using Pierce BCA protein assay kits (Thermo Fisher, Waltham, MA). Sample prep was similar to whole cell lysates after enriched synaptosome isolation.

### 2.8 Antibodies

Primary antibodies were rabbit GluN2B total, GluN2A total, pGluN2B (Tyr1472) pGluN2A (Tyr1246 and Tyr1325), CAMKII α/β(Thr286), Src (Tyr416), Src total, GAPDH and mouse CAMKIIα total (all from Cell Signaling Technology), pGluN2B (Ser1303) from Abcam, USA.

### 2.9 Statistical analysis

Statistical analysis was carried out using GraphPad Prism, version 7.04 (GraphPad Software, San Diego, CA) and StatView (Cary, NC). For all behavioral and molecular studies, statistical significance of group mean differences was measured by one-way analysis of variance (ANOVA), Two-way repeated measures ANOVA followed by Bonferroni correction test in behavioral studies. In molecular studies, Tukey post-hoc test was used after significant ANOVA. Immunoblotting graphs depict mean ± SEM. In all cases, *p* < 0.05 was defined as statistically significant.

## 3 Results

### 3.1 NMDAR canonical Src family kinase sites are differentially regulated by CCI-induced neuropathic pain and NYX-2925

**Fig. 1:**
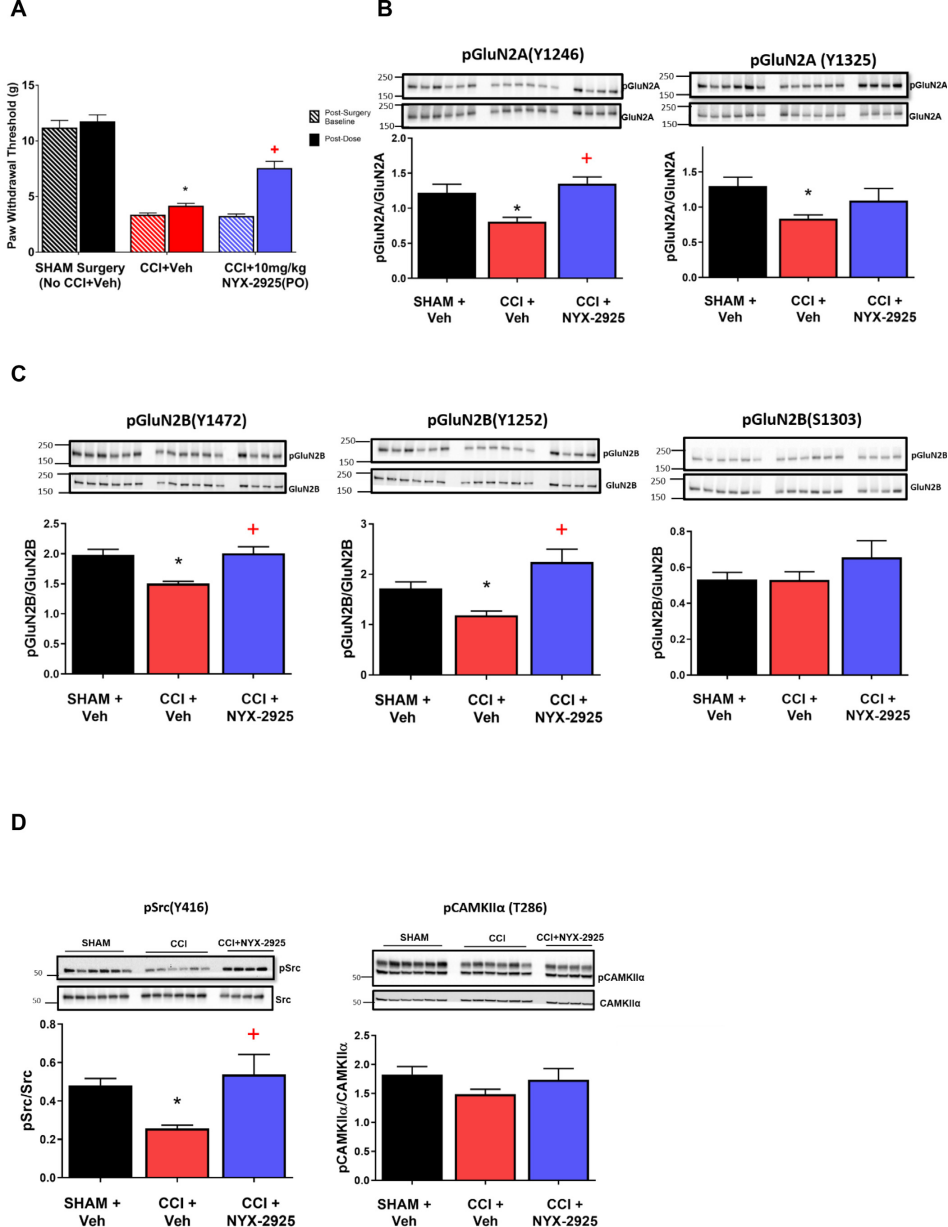
Src family kinase (SFK) may be critical for the analgesic effect of NYX-2925 in the rat CCI model of neuropathic pain. NYX-2925 administration restores pain induced decrease of Src-regulated proteins to baseline (SHAM) levels. A) Administration of 10 mg/kg NYX-2925 significantly increased paw withdrawal threshold (PWT) at 1 hr post-administration. mPFC tissues from these rats were collected and analyzed 24 hrs post oral dosing. Western blot for phosphorylated proteins B. GluN2A (Y1246 and Y1325) C. GluN2B (Y1472, Y1252 and S1303) D. Src (Y416) and CAMKIIα (T286) were normalized to their respective total protein levels in all groups. Significant down and restored changes were detected by one-way ANOVA followed by Tukey posthoc, p < 0.05. graphs depict means ± SEM; *p < 0.05 compared to SHAM + Veh, + p < 0.05 compared to CCI + Veh.

Previous studies demonstrate that oral administration of NYX-2925 at 10 mg/kg produced a rapid and long-lasting analgesic effect in neuropathic pain models (Ghoreishi-Haack et al., 2018). Approximately 2 weeks post CCI, rats with established and stable mechanical hypersensitivity were orally dosed with NYX-2925 (10 mg/kg) or vehicle and tested 1 hr post dosing. CCI rats showed mechanical hypersensitivity as demonstrated by low paw withdrawal thresholds (PWTs (less than 5 g)) in comparison with the SHAM group (Fig. 1A). Reproducing previous findings, oral administration of NYX-2925 (10 mg/kg) reversed mechanical hypersensitivity 1 hr post dosing when compared to the vehicle treated group F (5,60) = 45.67, p = 0.0012).

Pain induced changes in NMDAR levels and associated kinases in the mPFC were investigated in SHAM controls, vehicle-treated CCI rats, and NYX-2925-treated CCI rats. Several GluN2A and GluN2B phosphorylation sites that have been shown to either increase NMDA receptor at the synapse or increase receptor channel opening were evaluated (Carreño et al., 2011, Liu et al., 2008, Taniguchi et al., 2009). Two Src phosphorylation sites on GluN2A, Tyr1246 and Tyr1325, were assessed (Fig. 1B) and both phosphorylated sites were downregulated with CCI (p < 0.0001 for Tyr1246 CCI vs. SHAM; p = 0.0197 for Tyr1325 CCI vs. SHAM). NYX-2925 treatment restored the CCI-induced decrease to SHAM levels for phosphorylated Tyr1246 (p = 0.0003 CCI + NYX-2925 vs. CCI). Two Src kinase phosphorylation sites on GluN2B, Tyr1472 and Tyr1252, were also assessed and both phosphorylated Tyr1472 and Tyr1252 were decreased in neuropathic pain (p = 0.0004 for Tyr1472, CCI vs. SHAM; p = 0.0398 for Tyr1252, CCI vs. SHAM). NYX-2925 treatment restored levels of both phosphorylated Tyr1472 (p = 0.0005 for CCI + NYX-2925 vs. CCI) and Tyr1252 (p = 0.0002 for CCI + NYX-2925 vs. CCI) (Fig. 1C). Ser1303, a CAMKIIα phosphorylation site on GluN2B, was also assessed and phosphorylated Ser1303 showed no changes with CCI or after treatment with NYX-2925 (Fig. 1C).

Relevant kinase levels were also assessed. Phosphorylated Src (Tyr416), CAMKIIα (Thr286), were evaluated for changes in expression with CCI and after administration of NYX-2925 (Fig. 1D). A significant decrease in expression of phosphorylated Src was also seen (p = 0.0005 for pSrc CCI vs. SHAM) in the CCI model and NYX-2925 treatment, restored the levels to SHAM conditions (p = 0.0001 for CCI + NYX-2925 vs. CCI). There was no significant decrease in expression of phosphorylated CAMKIIα seen in CCI animals and the administration of NYX-2925 did not impact expression of CAMKIIα. Overall, these data suggest that NYX-2925 activity may be mediated through Src and not CAMKII family kinase dependent processes.

### 3.2 NYX-2925 specifically restores expression of phosphorylated synaptic proteins in the prelimbic PFC in the CCI model of neuropathic pain

**Fig. 2:**
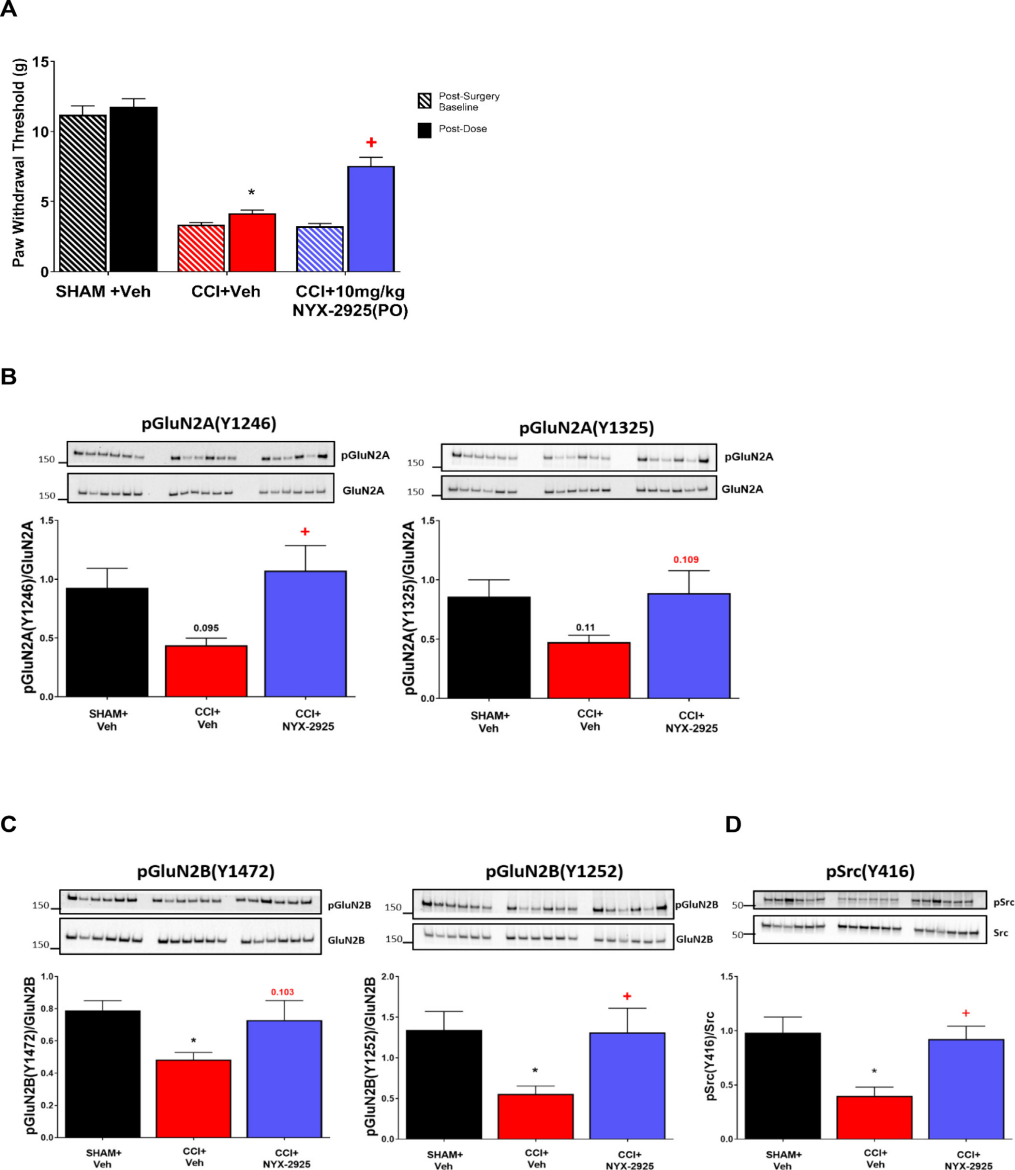
NYX-2925 specifically restores synaptic proteins in the prelimbic PFC of CCI rat model of neuropathic pain. A. NYX-2925 administration restores pain induced decrease of synaptic Src-regulated proteins to baseline (SHAM) levels. A) *.* Administration of 10 mg/kg NYX-2925 significantly increased paw withdrawal threshold (PWT) at 1hr post-administration. Enriched synaptosomal fractions of mPFC tissues from behavioral study above, were isolated and analyzed at 24 h post oral dosing. B. GluN2A (Y1246 and Y1325) C. GluN2B (Y1472 and Y1252).D. Src (Y416). Phosphorylated proteins were normalized to their respective total proteins. N = 12/group, significant down and restored changes were detected by one-way ANOVA followed by Tukey posthoc, p < 0.05. Graphs depict means ± SEM. *p < 0.05 compared to SHAM + Veh, + p < 0.05 compared to CCI + Veh.

To replicate and confirm the above findings with Src and evaluate the role of NYX-2925 in restoring pain-induced Src signaling specifically at the synapse, a separate group of CCI animals was used. Treatment with NYX-2925 (10 mg/kg) again produced an analgesic effect 1 hr post dosing when compared to vehicle group (F (2, 41) = 56.66, *p* < 0.0001, Fig. 2A).

To evaluate protein expression at the synapse, the mPFC was dissected and enriched synaptosomes were extracted. Like what was seen in whole cell lysates, the Src phosphorylation sites on GluN2A were downregulated in the synaptosome fraction of CCI animals, with both phosphorylated Tyr1246 (p = 0.095; CCI vs. SHAM) and Tyr1325 (p = 0.1102, CCI vs. SHAM) showing a trend toward a decrease under CCI compared to SHAM. Administration of NYX-2925 restored phosphorylated Tyr1246 (p = 0.0228; CCI + NYX-2925 vs. CCI) back to SHAM levels and showed a trend towards restoration to SHAM levels with Tyr1325 (p = 0.1091; CCI + NYX-2925 vs. CCI) (Fig. 2B). The Src phosphorylation sites on GluN2B, phosphorylated Tyr1252 (p = 0.0237; CCI vs. SHAM) and phosphorylated Tyr1472 (p = 0.033; CCI vs. SHAM) were also downregulated in the mPFC of CCI animals (Fig. 2C). NYX-2925 restored phosphorylated GluN2B Tyr1252 (p = 0.0414; CCI + NYX-2925 vs. CCI) to SHAM levels with a trend toward restoration seen with phosphorylated Tyr1472 (p = 0.1029; CCI + NYX-2925 vs. CCI) (Fig. 2C). Phosphorylated Src was also decreased in the CCI condition (p = 0.0036; CCI vs. SHAM). NYX-2925 administration restored phosphorylated Src levels back to SHAM levels (p = 0.0090; CCI + NYX-2925 vs. CCI) (Fig. 2D).

### 3.3 SFK inhibition in the prelimbic mPFC prevents the analgesic effect of NYX-2925 in CCI neuropathic pain rats

**Fig. 3:**
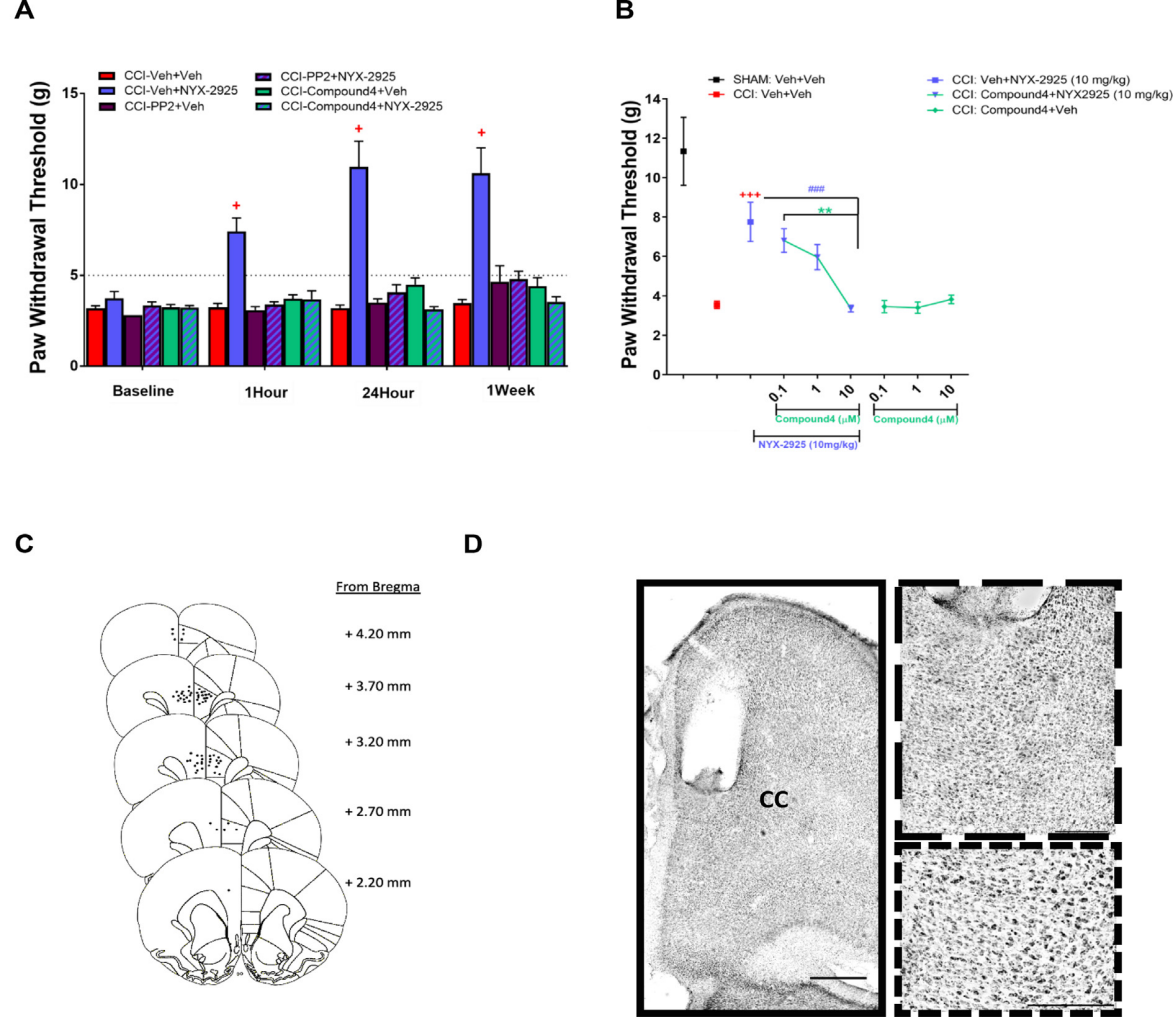
SFK inhibition in the prelimbic mPFC prevents the analgesic effect of NYX-2925 in CCI neuropathic pain rats. A. Direct mPFC infusion of Src Kinase inhibitors(SFKi) reverses the analgesic effect of NYX-2925 in the CCI model of neuropathic pain*.* Src family kinase inhibitors (10 µM, compound 4 or PP2) were infused in the mPFC for 20 min prior to NYX-2925 (10 mg/kg, PO) and tested for mechanical hypersensitivity 1, 24 hrs, and 1 wk post-dose. A two way repeated measures ANOVA that pretreatment with PP2 or Compound 4 blocked the analgesic effect of NYX-2925 at all time points (NYX-2925: F(1,28) = 44.678 p < 0.0001; SFKi: F(2,28) = 30.671p < 0.0001; Time: F(3,28) = 21.833) p < 0.0001; interaction: F(6,84) = 9.95) p < 0.0001. N = 6–7 animals per group. + p < 0.05 compared to CCI. B. Direct infusion of Compound4, a specific Src-Kinase inhibitor, into the mPFC 20 mins prior to NYX-2925 (10 mg/kg, PO) blocked the analgesic effect of NYX-2925 in a dose-dependent manner. *N =* 6–8 animals per group. + p < 0.05 compared to CCI, # p < 0.05 compared to CCI + NYX-2925, *p < 0.05 compared to 0.1 μM Compound 4 + NYX-2925. C. Placement of cannula tips in the medial prefrontal cortex of rats in the Src inhibition behavior study. The location of the placements ranged from 2.2 to 4.2 mm anterior to bregma. D. Nissl stained image showing cannula placement in the mPFC. Visual inspection of Nissl staining for chromatolysis and microglial activity directly below the infusion site showed an abundance of healthy appearing neurons. Bar = 250 µm. Individual groups- Surgery: mPFC infusion + Oral Dosing.

To evaluate the dependence of NYX-2925 analgesic activity on Src dependent NMDAR activation in the prelimbic mPFC, inhibitors of Src activation were administered directly onto the mPFC just prior to oral administration of NYX-2925. Two Src activation inhibitors were tested, a widely used, but non-selective Src family kinase (SFK) activation inhibitor-PP2, as well as a specific Src activation inhibitor - Compound 4 (KB SRC 4) (Brandvold et al., 2012). PP2 has a well described dose response – 10uM is the dose that is known to inhibit Src phosphorylation/activation in the mPFC (Barry and McGinty, 2017). Compound 4 has been shown to lead to the same level of phosphorylated Src inhibition as PP2 in an in vitro model at a 10uM concentration level (Brandvold et al., 2012), therefore a 10uM concentration of Compound 4 was also tested in the first animal study (Fig. 3). Rats underwent CCI surgery with bilateral mPFC cannulation immediately after nerve injury. The impact of bilateral infusion of 0.5 µL of PP2 (10 µM), Compound 4 (10 µM), or Vehicle (0.1% DMSO in double filtered PBS) on NYX-2925 was assessed 1hr, 24 hrs and 1 wk post oral NYX-2925 or vehicle administration. Oral administration of 10 mg/kg NYX-2925 with vehicle in the guide cannulae produced a significant analgesic effect at 1hr (*p* 0.0219); 24 hrs (*p* < 0.0289) and 1 wk (*p* 0.0375) post-dosing (Fig. 3A). The analgesic effect of oral NYX-2925 was blocked by bilateral mPFC infusion of either 10 µM PP2 (p < 0.0283; NYX-2925 + vehicle vs. NYX-2925 + PP2) or 10 µM Compound 4 (p < 0.0281; NYX-2925 + vehicle vs. NYX-2925 + Compound 4) 1 h post-NYX-2925 administration. Similarly, The blockade of NYX-2925 analgesic effect by bilateral mPFC infusion of either 10 µM PP2 (p < 0.0378, p < 0.0696 ; NYX-2925 + vehicle vs. NYX-2925 + PP2) or 10 µM Compound 4 (p < 0.0277; p < 0.0376 NYX-2925 + vehicle vs. NYX-2925 + Compound 4) was maintained at 24hrs and up to 1 week respectively. Bilateral infusion of either PP2 or Compound 4 alone did not have an impact on mechanical hypersensitivity in CCI animals at any timepoint, when tested 1 h after oral NYX-2925 or vehicle administration (Fig. 3A).

To confirm the effect of the specific Src activation inhibitor Compound 4 as well as to better understand the dose response, a separate set of CCI rats was administered Compound 4 over a wide dose range (0.1–10 µM; Fig. 3B). Like the previous experiment, administration of vehicle into the guide cannulae had no effect on the analgesic activity of oral NYX-2925 (10 mg/kg) 1hr post-dosing (p = 0.0025). Animals administered Compound 4 (0.1–10 µM) into the mPFC had no change in the pain response following oral administration of vehicle. mPFC administration of Compound 4 prior to oral administration of NYX-2925 blocked the 1hr analgesic activity of NYX-2925 in a dose dependent manner with a complete block of analgesic activity seen with 10 µM Compound 4 (p = 0.0033 Compound 4 in the mPFC + oral NYX-2925 vs. vehicle in the mPFC + oral NYX-2925). Administration of either 0.1 or 1 µM compound 4 into the mPFC had no effect on analgesic activity of oral NYX-2925. Fig. 3C and D are a representation of mPFC cannula placements for all studies, and a Nissl stain confirming the location.

### 3.4 SFK inhibition prevents NYX-2925 induced upregulation of synaptic phosphorylated Src and Src-dependent NMDAR phosphorylation sites

**Fig. 4:**
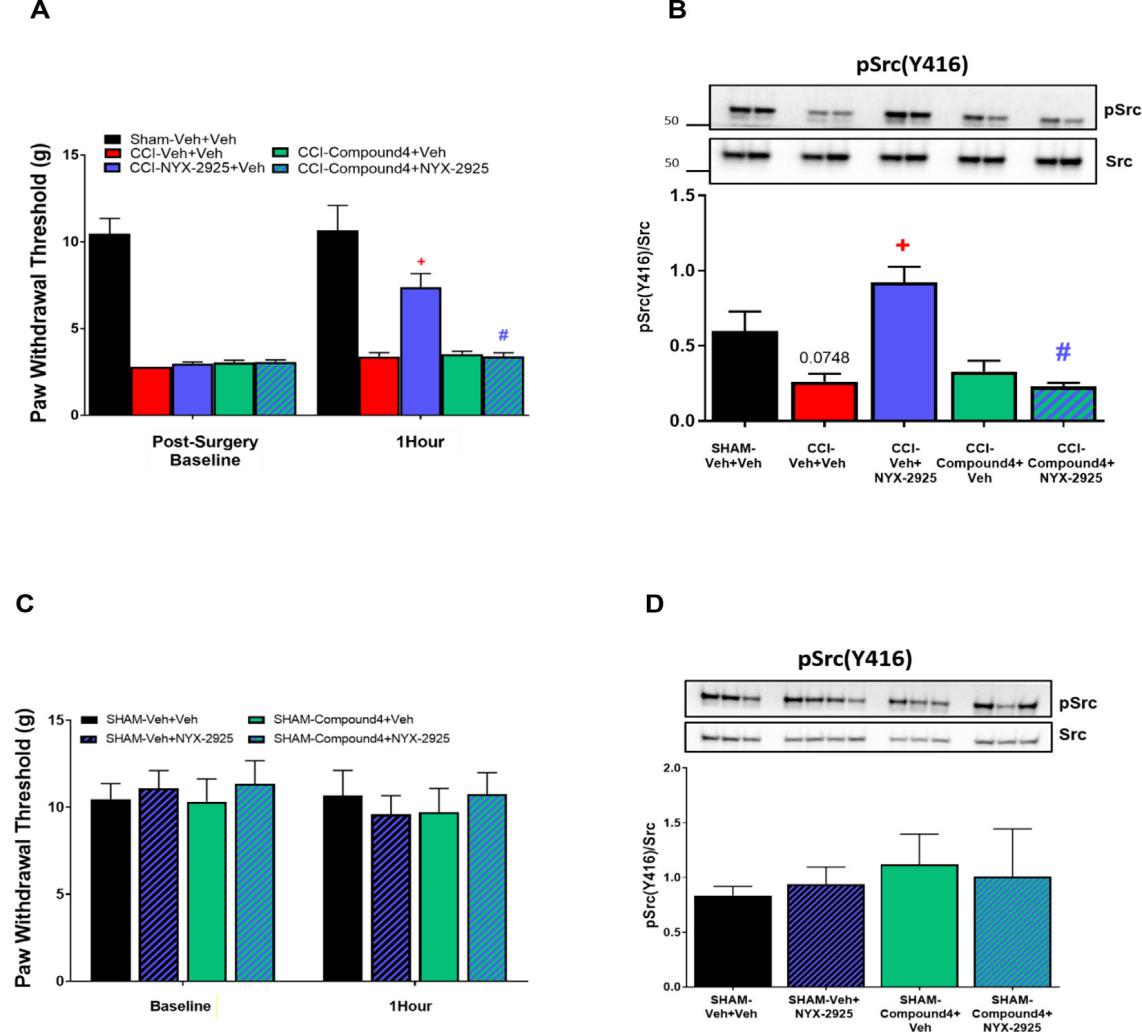
SFK inhibition prevents NYX-2925 induced upregulation of synaptic phosphorylated Src and Src-dependent NMDAR phosphorylation sites. Inhibition of Src kinase, prevents the NYX-2925-driven upregulation of phosphorylated Src A. Direct mPFC infusion of Compound4 reverses the analgesic effect of NYX-2925. Compound 4 was infused in the mPFC for 20 min prior to NYX-2925 (10 mg/kg, PO) and mechanical hypersensitivity 1 hr post-dose was tested. A two way ANOVA shows that pretreatment with Compound 4 blocked the analgesic effect of NYX-2925 1 h post dose (NYX-2925: F(1,26) = 20.187 p < 0.0001; Compound 4 effect F(1,26) = 18.374 p < 0.0002; interaction F(1,26) = 19.212 p < 0.0002). For signaling analysis, mPFC samples were collected 24 hrs post-dose. Enriched synaptosomes were prepared from the mPFC samples collected B. Western blots for phosphorylated Src (pY416) were analyzed. C. Direct infusion of Compound4, a specific Src-Kinase inhibitor, into the mPFC 20 mins prior to NYX-2925 (10 mg/kg, PO), showed no effect in SHAM animals. D. Western blots for phosphorylated Src (pY416) in SHAM rats were analyzed. Values are expressed as means ± SEM and were analyzed by one-way ANOVA followed by Tukey posthoc. N = 6–7 animals per group. (p * <0.05 compared to SHAM, +<0.05, compared to CCI, # <0. 05 compared to CCI + NYX-2925). Individual groups- Surgery: mPFC infusion + Oral Dosing.

Compound 4 is a more specific Src inhibitor than PP2 so to understand the impact of phosphorylated Src inhibition on NYX-2925-induced changes of synaptic proteins in CCI, Compound 4 was used. Analgesic activity of NYX-2925 (10 mg/kg) was confirmed in a separate set of CCI animals 1hr post-dosing (*p* < 0.0001) (Fig. 4). Reproducing the prior experiments, bilateral infusion of Compound 4 (0.5 µL of 10 µM) into the mPFC, 20 mins prior to oral NYX-2925 dosing, blocked the analgesic effect of NYX-2925 when tested 1 h later (*p* < 0.0001) (Fig. 4A). Animals were sacrificed and brain dissected 24 hrs post-dosing with NYX-2925. Samples were collected around the cannula tract in the mPFC and synaptosomal fractions were isolated. As shown previously, phosphorylated Src was decreased under CCI conditions compared to SHAM (p = 0.0386; CCI vs. SHAM) and systemic NYX-2925 administration restored phosphorylated Src levels (p = 0.0004; CCI + NYX-2925 vs. CCI). Microinjection of Compound 4 into the mPFC of CCI animals showed no effect on phosphorylated Src compared to CCI + Veh alone. However, microinjection of Compound 4 into the mPFC blocked NYX-2925’s mediated restoration of Src in CCI animals (p = 0.0003, CCI + NYX-2925 + Compound4 vs. CCI + NYX-2925 Fig. 4B). Bilateral infusion of Compound 4 in the mPFC did not have an impact on mechanical hypersensitivity in SHAM animals when tested 1 h after oral NYX-2925 or vehicle administration (Fig. 4C). Additionally, oral dosing of NYX-2925 had no effect in SHAM rats, confirming previous findings (Ghoreishi-Haack et al., 2018). Biochemical analysis of the SHAM samples from Fig. 4C showed no difference in protein expression of phosphorylated Src (Fig. 4D).

## 4 Discussion

NYX-2925 is a NMDAR modulator that alleviates neuropathic pain in several animal models. NYX-2925 enhances NMDAR current and facilitates LTP (Khan et al., 2017), and direct injection of NYX-2925 into the mPFC, alleviates neuropathic pain while intrathecal administration has no effect (Ghoreishi-Haack et al., 2018). In the present study, the CCI model of neuropathic pain was used to investigate the effect of NYX-2925 on NMDAR-mediated signaling in the mPFC in order to identify the underlying mechanism by which NYX-2925 alleviates neuropathic pain. NMDARs are known to play an important role in neuropathic pain (Wu and Zhuo, 2009, Zhou et al., 2011). The regulation of NMDAR channel activity, trafficking and stabilization of receptors at the surface by Src (Goebel-Goody et al., 2009, Salter et al., 2009, Yu et al., 1997) and CAMKII18, has been implicated in the maintenance of neuropathic pain (Katano et al., 2011). However, the vast majority of studies assessing the role of Src and CamKII on neuropathic pain regulation have focused on their impact in the spinal cord (Liu et al., 2008, Zhou et al., 2017). Given our previous data showing the importance of mPFC in the pain alleviation effect of NYX-2925, we focused our study on processes occurring in the mPFC.

In the mPFC, studies have shown impaired glutamatergic signaling along with an overall dampening of mPFC activity with chronic neuropathic pain (Ji and Neugebauer, 2011, Kelly et al., 2016). Additionally, optogenetic activation of the region results in an alleviation of pain (Ji and Neugebauer, 2011, Wang et al., 2015), suggesting that dampening of mPFC activity may be directly contributing to the pain response. This is in contrast to the spinal cord, where studies in animals with neuropathic pain have shown a hyperactivation of glutamatergic signaling and an increase in Src activity (Hildebrand et al., 2016, Liu et al., 2008), with pain alleviation achieved through NMDAR antagonism approaches (Chaplan et al., 1997, Zhou et al., 2011). Our studies show that phosphorylated Src as well as Src phosphorylation sites on GluN2A and GluN2B, known to be upregulated by neuropathic pain in the spinal cord, were decreased in the mPFC following CCI. Given the opposing disruption of glutamatergic signaling seen with neuropathic pain in the spinal cord vs. the mPFC, it is not surprising that our data showed a downregulation of Src activity in the mPFC. Src-mediated NMDAR activation appears to play an important role in pain regulation in both the spinal cord and brain, albeit through contrasting directions.

An analgesic dose of NYX-2925 (10 mg/kg, PO) restored the pain induced decrease of phosphorylated levels of Src and Src mediated NMDAR phospho-proteins in both whole cell lysates and enriched synaptic fractions. These data suggest that reactivation of NMDAR signaling in the mPFC may be important for the alleviation of pain seen after oral dosing of NYX-2925. NYX-2925 did not show any added effect on phosphorylated Src in SHAM animals and thus no change in paw withdrawal threshold. Src activation inhibitors were administered alongside NYX-2925 to better assess the dependence of NYX-2925 analgesic activity on Src-mediated processes. PP2 is a well-studied SFK inhibitor (Barry and McGinty, 2017, Liu et al., 2008), but has several off-target actions other than specific Src kinase inhibition (Brandvold et al., 2012). For this reason, we also assessed Compound4, a highly selective Src-kinase activation inhibitor (Brandvold et al., 2012). Under CCI conditions where NYX-2925 rapidly drives the phosphorylation/activation of Src, both Src kinase activation inhibitors blocked the analgesic effect of NYX-2925 in CCI animals as early as 1 h and up to 1 week post dosing. This shows that the analgesic effect of NYX-2925 in the treatment of neuropathic pain may be through enhancement and regulation of Src-dependent NMDAR signaling cascades in the mPFC. Neither Src activation inhibitors alone had any effect on neuropathic pain in CCI animals after direct injection into the mPFC, likely because Src kinase activity in the mPFC was already downregulated in the CCI model of neuropathic pain. Similarly, Compound 4 injection into the mPFC did not increase the pain response in SHAM animals, when measured 1 h after treatment. This is not surprising given that Src is not constitutively activated in neurons in a non-disease state (Marin et al., 2010). One would expect that Src activation would need to be blocked for an extended duration of time to see an effect on overall Src levels and the pain response. The window in which Compound 4 was administered as well as when these measurements were taken relative to Compound 4 administration, was likely not sufficient to detect an effect of Compound 4 on pain in SHAM rats.

CAMKII is another kinase that specifically regulates GluN2B and has a role in the maintenance of neuropathic pain (Hunt and Castillo, 2012, Qian et al., 2018). Unlike what was found with Src, phosphorylated CAMKII levels showed no change in the mPFC of CCI animals, which is different from what has been found to occur in the spinal cord (Katano et al., 2011, Qian et al., 2019). Additionally, levels of the GluN2B CAMKII phosphorylation site (Ser1303) were not changed in the mPFC, of the CCI model of neuropathic pain. Treatment of CCI animals with NYX-2925 reduced neuropathic pain but had no impact on phosphorylated CAMKII in the mPFC, suggesting that in the rat CCI model, CAMKII activation may not be important for the analgesic effect of NYX-2925.

Changes in central brain activity and circuitry are increasingly recognized as key drivers in the development and maintenance of chronic neuropathic pain (Baliki et al., 2012, Bushnell et al., 2013, Mutso et al., 2012). NYX-2925 is an NMDAR modulator that has shown analgesic effect in a number of neuropathic pain models after systemic (oral) or direct mPFC administration in rats (Ghoreishi-Haack et al., 2018). During neuropathic pain, NMDAR-mediated plasticity is heightened in the spinal cord and dampened in the mPFC. While most compounds developed for the treatment of neuropathic pain target pain processing in the spinal cord, the specific brain-mediated activity of NYX-2925 may make the compound uniquely positioned as a novel therapeutic in this space.

**Fig. 5:**
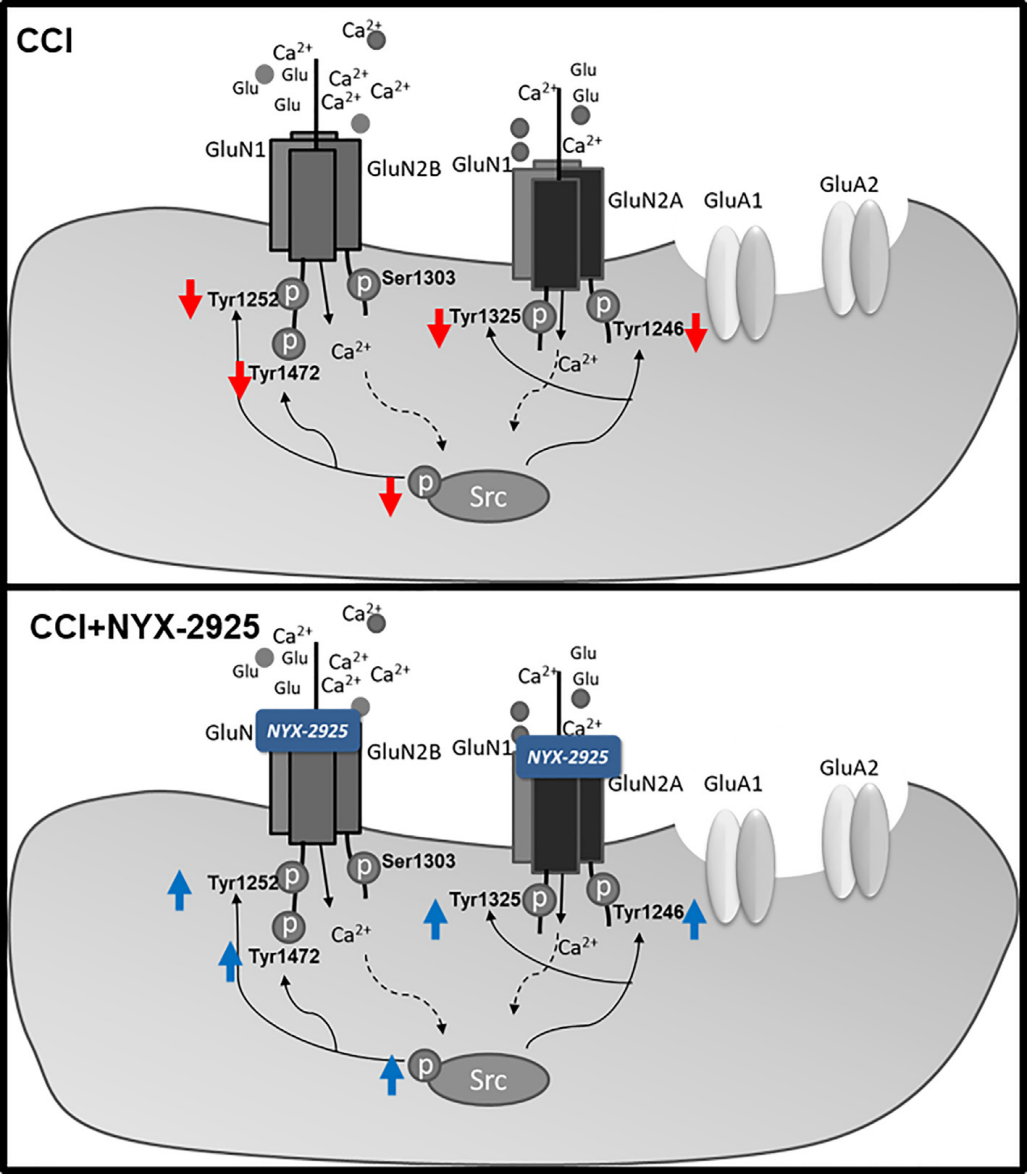
Proposed mechanism of the analgesic effect of NYX-2925 in the mPFC of CCI and CCI + NYX-2925 rats. The CCI model of neuropathic pain is characterized by a significant decrease in several phosphorylated GluN2A and GluN2B receptors and the downstream regulator Src kinase in the mPFC. We postulate that addition of NYX-2925 enhances calcium influx which eventually results in the activation of Src. Src then activates several GluN2A and GluN2B phosphorylation sites, resulting in a positive feedback loop, stabilizes NMDAR at the surface and leads to the alleviation of pain.

Our present findings suggest that a downregulation of Src activity in the mPFC occurs with neuropathic pain and that the analgesic effect of NYX-2925 may be through a restoration of Src activity and overall NMDAR-mediated plasticity in the brain (Fig. 5). We hypothesize that after oral administration of NYX-2925, the compound binds to NMDARs (Khan et al., 2017), initiating a signaling cascade that ultimately activates Src. Phosphorylated Src then activates sites on GluN2A and GluN2B that are known to stabilize NMDAR receptors at the synapse and likely underlies NYX-2925’s long lasting effect on plasticity (Ghoreishi-Haack et al., 2018, Khan et al., 2017). This positive feedback elicited by NYX-2925 ultimately results in the alleviation of neuropathic pain. Src-mediated signaling can be activated by both ionotropic (Salter and Kalia, 2004) and metabotropic NMDAR signaling (Weilinger et al., 2016) through the NMDAR. Thus, it is intriguing that we have previously shown that NYX-2925 can dose-dependently increase intracellular calcium or signal via NMDAR-dependent metabotropic mechanisms in vitro (Bowers et al., 2019). Thus, future studies should examine the relevance of these two modes of NYX-2925 action in alleviating chronic pain.

Overall, our data supports continued development of NYX-2925 for the treatment of neuropathic pain and suggests that NYX-2925 is likely to be more effective in pain populations where the disease involves changes in central regions such as the mPFC, which is supported by recent clinical trial data (Torri et al., 2019).

## Declaration of Competing Interest

G. Morrison, M.N Asiedu, J.M Priebe, N. Ghoreishi-Haack, M.S Bowers, A.L Barth, and C.N. Cearley are current employees and J. Dunning, R.A Kroes, and J.R. Moskal are former employees of Aptinyx, Inc. and have stock/ownership in Aptinyx, Inc.
